# Artificial intelligence-based, semi-automated segmentation for the extraction of ultrasound-derived radiomics features in breast cancer: a prospective multicenter study

**DOI:** 10.1007/s11547-024-01826-7

**Published:** 2024-05-09

**Authors:** Tommaso Vincenzo Bartolotta, Carmelo Militello, Francesco Prinzi, Fabiola Ferraro, Leonardo Rundo, Calogero Zarcaro, Mariangela Dimarco, Alessia Angela Maria Orlando, Domenica Matranga, Salvatore Vitabile

**Affiliations:** 1 Department of Biomedicine, Neuroscience and Advanced Diagnostics (BiND), University of Palermo, Palermo, Italy; 2 Institute for High-Performance Computing and Networking (ICAR-CNR), Italian National Research Council, Palermo, Italy; 3 Department of Information and Electrical Engineering and Applied Mathematics (DIEM), University of Salerno, Fisciano, SA Italy; 4 Breast Unit, Fondazione Istituto “G. Giglio”, Cefalù, PA Italy; 5 Department of Health Promotion, Mother and Child Care, Internal Medicine and Medical Specialties (ProMISE), University of Palermo, Palermo, Italy; 6 Department of Computer Science and Technology, University of Cambridge, Cambridge, UK

**Keywords:** Ultrasound, Breast cancer, Artificial intelligence, Machine learning

## Abstract

**Purpose:**

To investigate the feasibility of an artificial intelligence (AI)-based semi-automated segmentation for the extraction of ultrasound (US)-derived radiomics features in the characterization of focal breast lesions (FBLs).

**Material and methods:**

Two expert radiologists classified according to US BI-RADS criteria 352 FBLs detected in 352 patients (237 at Center A and 115 at Center B). An AI-based semi-automated segmentation was used to build a machine learning (ML) model on the basis of B-mode US of 237 images (center A) and then validated on an external cohort of B-mode US images of 115 patients (Center B).

**Results:**

A total of 202 of 352 (57.4%) FBLs were benign, and 150 of 352 (42.6%) were malignant. The AI-based semi-automated segmentation achieved a success rate of 95.7% for one reviewer and 96% for the other, without significant difference (*p* = 0.839). A total of 15 (4.3%) and 14 (4%) of 352 semi-automated segmentations were not accepted due to posterior acoustic shadowing at B-Mode US and 13 and 10 of them corresponded to malignant lesions, respectively. In the validation cohort, the characterization made by the expert radiologist yielded values of sensitivity, specificity, PPV and NPV of 0.933, 0.9, 0.857, 0.955, respectively. The ML model obtained values of sensitivity, specificity, PPV and NPV of 0.544, 0.6, 0.416, 0.628, respectively. The combined assessment of radiologists and ML model yielded values of sensitivity, specificity, PPV and NPV of 0.756, 0.928, 0.872, 0.855, respectively.

**Conclusion:**

AI-based semi-automated segmentation is feasible, allowing an instantaneous and reproducible extraction of US-derived radiomics features of FBLs. The combination of radiomics and US BI-RADS classification led to a potential decrease of unnecessary biopsy but at the expense of a not negligible increase of potentially missed cancers.

## Introduction

Breast cancer is the most common cancer in women overall and the fifth leading cause of cancer mortality worldwide, with 685,000 deaths in 2020 [1]. Because of its incidence and clinical impact, early and accurate cancer detection and characterization is of utmost importance to reduce breast cancer mortality and to improve the quality of life, by limiting the need of neoadjuvant or adjuvant therapies and allowing patients to be treated with breast conserving surgery [2].

In recent years, artificial intelligence (AI) has been introduced in the clinical field, enabling useful support of decision-making process of clinicians [3, 4]. Under the assumption that biomedical images contain information not visible by the naked human eye but still detectable via quantitative analysis, radiomics aims to extract the information 'hidden' behind the pixels, by means of mathematically defined features able to model the texture/patterns of the image [5, 6]. This is of relevance, considering that the reported 16% of breast cancers that are missed by radiologists likely reflect limitations in image perception by the human eye [7]. In particular, tree ensemble algorithms, such as XGBoost, have proven effective for classification in small datasets [8, 9] and widely used in radiomic works [10, 11].

Radiomics approach has been applied with encouraging results to X-ray-based techniques and MRI for breast cancer detection, treatment response assessment and disease progression monitoring [12, 13]. More recently, radiomics analysis has been applied to breast ultrasound (US), focusing either on the detection and characterization of breast cancer or on the prediction of biological behavior, nodal status and molecular subtypes [14–17]. Ciritis et al. evaluated a deep convolutional neural network (dCNN) based on 1019 US images from 582 patients for detection, highlighting and classification breast lesions mimicking human decision making, with a reported AUC on the internal dataset of 83.8 (external 96.7) for the dCNN and 84.6 ± 2.3 (external 90.9 ± 2.9) for two independent experienced radiologists [18]. A multicenter study reported that a dCNN based on 4828 US images from 1275 patients with primary breast cancer enabled the assessment of breast cancer molecular subtypes with a diagnostic accuracy ranging from 80.07 to 98.83% in two different test cohorts [17].

Nevertheless, radiomics is a complex, multistep process including image acquisition and segmentation, feature extraction and selection, classifier modeling and validation [19]. In particular, the task of segmentation is crucial to obtain meaningful features but, at the same time, it is prone to error and is very time-consuming, especially when it is manually performed. Within this scenario, recent advances in artificial intelligence (AI) have made possible breast masses detected at ultrasound to be automatically contoured and characterized according to the BI-RADS US lexicon [20, 21].

We hypothesized that an artificial intelligence-based semi-automated lesion segmentation may speed the process of an efficient and reproducible extraction of ultrasound-derived radiomics features. Secondly, on the basis of the extracted features, we developed and tested a specific radiomics model for the characterization of focal breast lesions (FBLs).

## Materials and methods

### Study design, patients and imaging data selection

Institutional ethical committee approval was obtained, and full written informed consent was obtained for this prospective study. Our study complied with the terms of the Declaration of Helsinki [22].

**Fig. 1:**
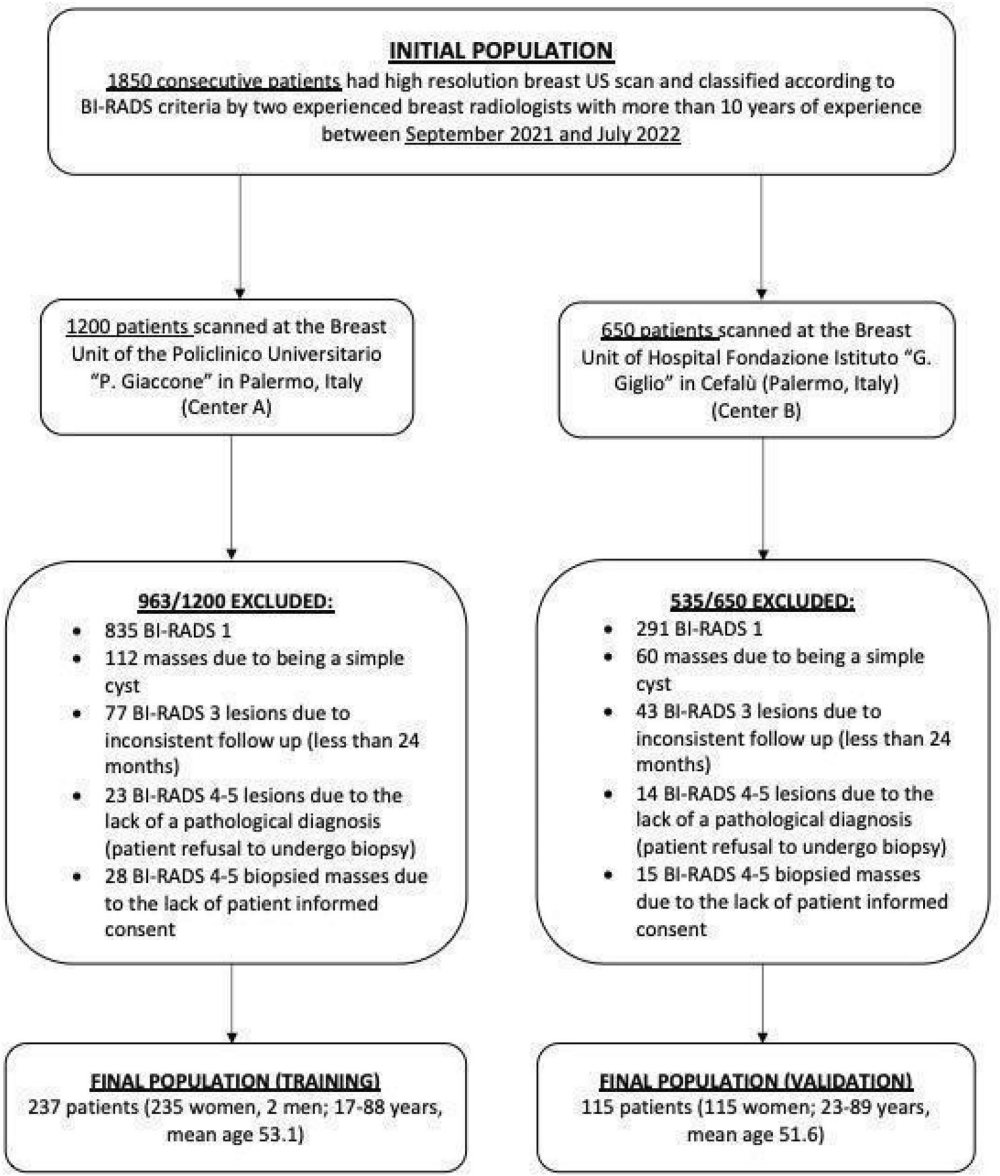
Flowchart for the patient selection

Figure 1 shows the detailed flowchart for the patient selection. Between September 2021 and July 2022, 1850 consecutive patients underwent high resolution breast US (HRUS) in two different Breast Units. One thousand two hundred patients were scanned at the Breast Unit of the Policlinico Universitario ‘P. Giaccone’ in Palermo, Italy (Center A) and 650 patients underwent HRUS at the Breast Unit of the Hospital Fondazione Istituto ‘G. Giglio’ in Cefalù (Palermo, Italy) (Center B).

Two experienced breast radiologists (one for each center, more than 30 years of experience in breast imaging, including ultrasound) performed HRUS by means of two identical ultrasound units, each provided with the same 3–12 MHz linear transducer (RS85 Samsung Medison, Co. Ltd.). The two radiologists classified HRUS findings according to US BI-RADS criteria [23]. All the US BI-RADS 3, 4 and 5 lesions were included. In patients with multiple lesions, only the one best satisfying the inclusion criteria was selected. All the pertaining images were stored on the hard disks of the US equipment and on the respective institutional PACS in DICOM format.

In both centers, indications for breast US included: (1) a palpable mass detected on physical examination; (2) dense breasts; (3) detected lesions from adjunct mammography examination; (4) patients with mastodynia; (5) young patients having family history or (6) in a follow-up for benign breast nodules (7) patients must not have undergone any intervention or surgery on lesions before US examination. Exclusion criteria included lack of adequate standard of reference (refusal to undergo biopsy or inconsistent follow-up).

### Standard of reference (SOR)

For the purpose of this study, US-guided core-biopsy was considered as SOR for all the FBLs classified as BI-RADS 4 or 5. For all lesions classified as BI-RADS 3, US findings at 6-, 12- and 24-month follow-up were considered as SOR. In particular, stability or size decrease during follow-up was considered typically benign US findings.

### Radiomics analysis

**Fig. 2:**
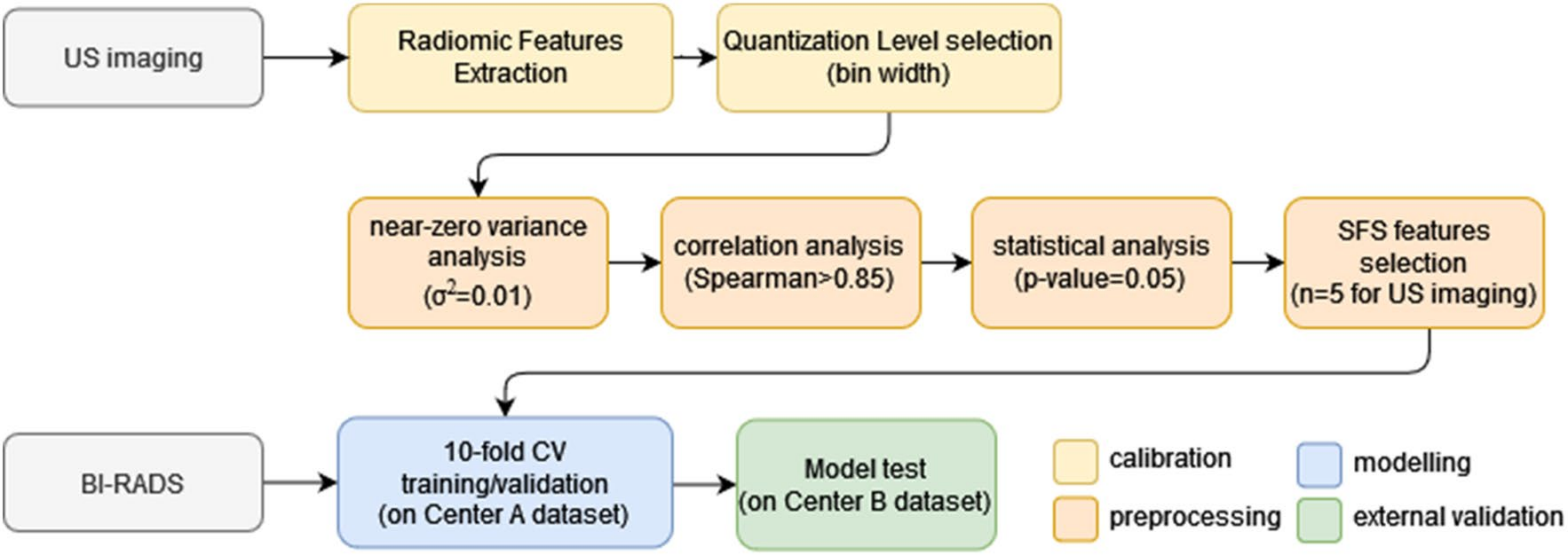
The flow diagram of the implemented methodology for radiomics analysis

The radiomics process involves several steps: ROI segmentation, feature extraction, features selection, model building and validation. In Fig. 2, the overall flow diagram of the implemented methodology is reported.

The information and metadata—contained in the DICOM header—about patients involved in that study were deleted before starting the analysis. In particular, using the MATLAB IDE a custom-made tool was implemented, able to load the ultrasound images, extract the segmentation mask and to convert them (image and corresponding mask) into NIFTI (.nii) format. During this conversion, all metadata (e.g., patient name, date of birth, etc.) were discarded. Only metadata related to the pixel matrix (e.g., pixel spacing, pixel size, etc.) were retained.

### Lesion segmentation

Concerning the segmentation, we used S-Detect™, a software commercially available and licensed for clinical use, installed and running on the Samsung RS85 ultrasound system [24]. In particular, S-Detect™ is an artificial intelligence software based on a convolutional neural network (Samsung Medison Co., Ltd, South Korea) that has been trained to classify focal breast lesions according to ACR US BI-RADS lexicon using over 10,000 breast scans against ‘gold standard’ biopsy assessments. By placing an operator-defined marker within the FBL, S-Detect™ automatically and instantaneously draws the contour of the lesion. The operator can either accept the automatically defined ROI or modify it manually. The radiologist was asked to take note of the acceptance or not of the suggested segmentation.

In this study, two radiologists extracted from each B-mode image the breast lesion by means of S-Detect™. To assess and validate the segmentation results, two additional experienced radiologists (one for each center, more than 5 years of experience in breast imaging, including ultrasound) reviewed all the stored images and selected the most representative US B-mode image of each FBL.

### Radiomic feature extraction and selection

Radiomic features were extracted from the segmented US images of both centers using PyRadiomics, a toolkit compliant with the Image Biomarkers Standardization Initiative [25]. PyRadiomics extracted a total a 102 unfiltered radiomic features, belonging to the following categories:

- Nine features belonging to shape 2D category, related to two-dimensional size and shape of the lesion, considering the segmentation mask
- Eighteen features belonging to first-order (FO) category: describe the voxel intensities distribution
- Twenty-four features belonging to gray-level co-occurrence matrix (GLCM) category, modeling spatial relationship between pixels, highlighting property of uniformity, homogeneity, randomness and linear dependencies [26]
- Sixteen features belonging to gray-level size zone matrix (GLSZM) category, describing intensity variations or the homogeneity distribution of regions [27]
- Sixteen features belonging to gray-level run length matrix (GLRLM) category, model the texture in a specific spatial direction [28]
- Fourteen features belonging to gray-level dependence matrix (GLDM) category, quantities dependencies among gray level of pixels [29]
- Five features belonging to neighboring gray tone difference matrix (NGTDM) category, referring to spatial relationship among pixels, approaching the human image perception [30].

Then, a preprocessing phase—aimed at obtaining a subset of robust, non-redundant and informative features—was performed [31, 32]. Successively, the sequential feature selector algorithm combined with the XGBoost classifier was used to select the most discriminating features. XGBoost is one of the most common tree ensemble algorithms, capable of minimizing the loss function of the model by adding weak learners using gradient descent (boosting ensemble method) [33].

### Training and external validation

The setup of the predictives models, based on a XGBoost classifier, trained/tested using a stratified tenfold CV procedure repeated 20 times on the Center A dataset. Two different input configurations were considered: (a) BI-RADS only and (b) BI-RADS + radiomic features. Figure 2 outlines the proposed approach for breast cancer characterization exploiting the BI-RADS and US-derived radiomic features. The main hyper-parameters used in the training phase are: num. of estimators: 100; learning rate: 0.3; booster: gbtree; objective function: binary logistic.

The external validation of predictive models was carried out on an external cohort consisting of the dataset from center B.

XGBoost model was used to classify ‘malignant’ *vs.* ‘benign’ FBLs. XGBoost is well established as a standard tool to process tabular data and improve performance over deep architectures [34]. The reason why we used shallow (not deep) machine learning techniques is twofold. The first is the need for explainability: shallow learning and explainable methods provide insights into the features driving their decisions, allowing clinicians to understand the reasoning behind the system’s recommendations [35]. The second is the training in small data scenarios: deep learning methods are well known to require huge amounts of data for training. The use of radiomic features enables the use of shallow learning methods, which remain attractive for training in small dataset scenarios.

### Clinical explanation of the predictive model

To implement the explainability, mandatory in clinical scenarios, the relative importance—by quantifying the Gini importance—was used to evaluate the weight of each feature in the final prediction [36].

### Statistical analysis

Statistical and computational analyses were performed using the MATLAB® R2020b environment (MathWorks, Natick, MA, USA). Continuous normally distributed variables were reported as mean and standard deviation (SD), while categorical variables were reported as number and percentage of patients with the specific characteristics.

For ‘Benign’ and ‘Malignant’ distribution comparisons, the nonparametric Wilcoxon rank-sum test (Mann–Whitney *U* test) was used, using a significance level of 0.05.

The used evaluation metrics were the accuracy, sensitivity, specificity, f1-score and AUROC, along with positive predictive value (PPV) and negative predictive value (NPV) to better investigate true positive and true negative rates, respectively [37].

For comparing matched samples (obtained by the two models, exploiting only BI-RADS and BI-RADS + radiomic), the nonparametric Wilcoxon signed-rank test on paired samples was used.

Finally, for comparing the diagnostic accuracy of BI-RADS and BI-RADS + Radiomics, the z test for proportions was used. *P* < 0.05 was chosen as the cutoff for statistical significance.

## Results

According to selection criteria, a total of 352 FBLs (size range: 3–90 mm; mean size ± SD: 14.9 ± 9.1 mm) were detected in 352 patients (350 women, 2 men; age range: 17–89 years; mean age ± SD: 52.6 ± 14.9 years). According to SOR, 202 out of 352 (57.4%) FBLs were benign, and 150 out of 352 (42.6%) were malignant (Table 1).

**Table 1:** Characteristics of 352 focal breast lesions according to SOR and enrollment center

Diagnosis	Center A	Center B	Total
*Malignant lesions*			
Invasive ductal carcinoma	70	33	103
Invasive lobular carcinoma	17	3	20
Other*	13	9	22
Total	100	45	145
*Tumor grade*			
G1	12	9	21
G2	51	24	75
G3	34	10	44
Total	97	43	140
*Molecular subtype*			
Luminal A/B	80	39	119
HER2 +	11	2	13
Triple negative	6	2	8
Total	97	43	140
*Benign lesions*			
Fibroadenoma	90	29	119
Complicated cyst	23	24	47
Phyllodes tumor	2	1	3
Other**	22	16	28
Total	137	70	207

^*^Ductal carcinoma in situ, metaplastic carcinoma, tubular carcinoma, primary angiosarcoma, malignant phyllodes tumor

^**^Fibrosis, radial scar, hamartoma, lipogranulomatosis, abscess

**Fig. 3:**
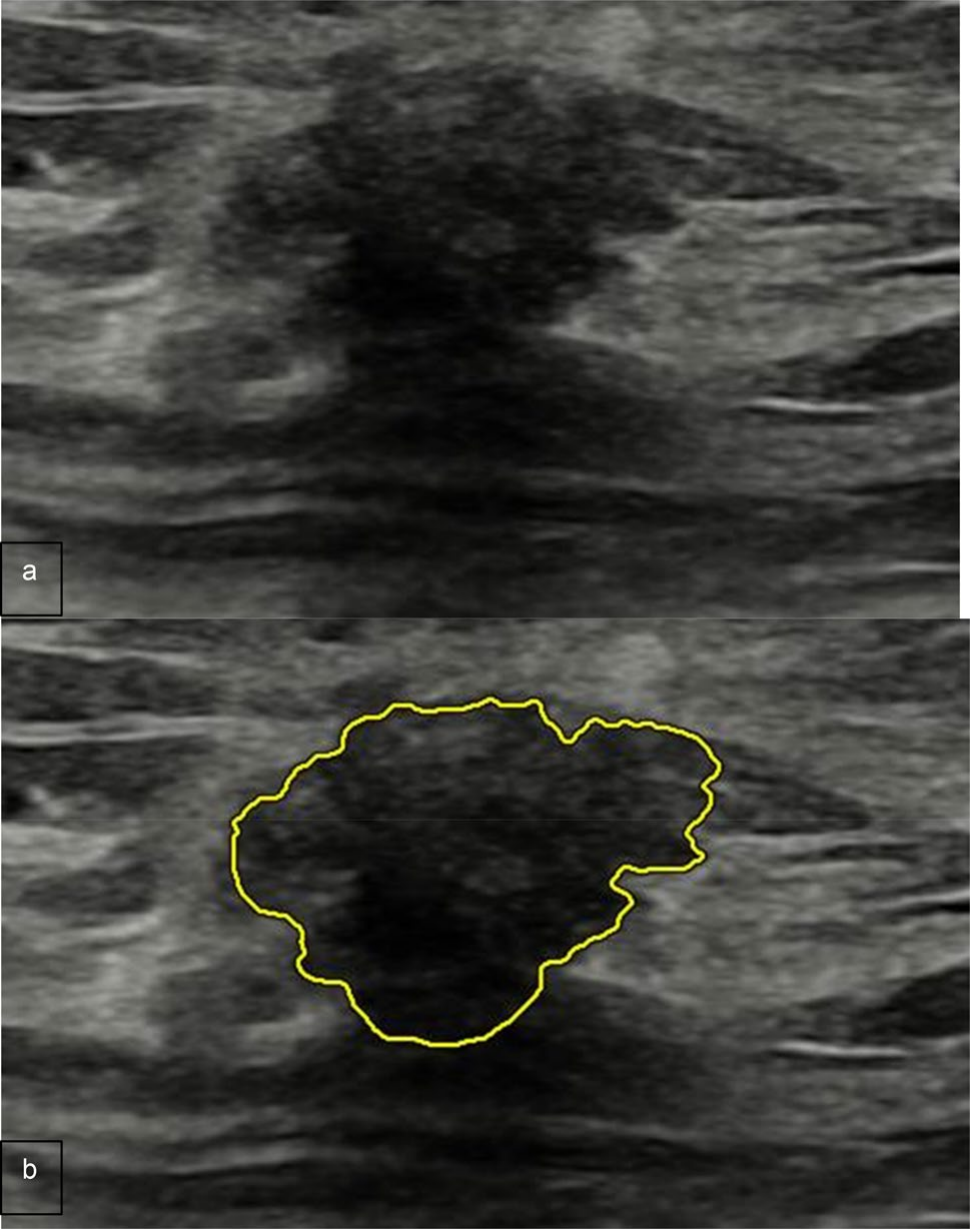
Luminal A invasive ductal carcinoma in a 53-year-old woman. **a** B-mode US shows a 1 cm hypoechoic focal breast lesion, irregular-shaped with indistinct and angulated margins, not parallel orientation and posterior shadowing. **b** AI-based semi-automated lesion segmentation. The lesion contour was eventually re-drawn by the radiologist manually

Table 2 details the acceptance rate of AI-based semi-automated lesion segmentation by two radiologists, which showed no statistically significant difference (*p* = 0.839). Fifteen (4.3%) and 14 (4%) of 352 AI-based semi-automated lesion segmentations were not accepted by the two reviewers, respectively. Among these unaccepted segmentations, 13 and 10 were malignant, respectively. All of these lesions showed a posterior acoustic shadowing at B-Mode US (Fig. 3).

**Table 2:** Acceptance rate of AI-based semi-automated segmentation of 352 focal breast lesions

	Total (352)	Benign (202)	Malignant (150)
Radiologist 1	337 (95.7%)	200 (99%)	137 (91.3%)
Radiologist 2	338 (96%)	198 (98%)	140 (93.3%)
*p*-value	0.850	0.411	0.515

### Radiomic features extraction and selection

A total of 103 unfiltered radiomic features were extracted from the US images, belonging to the following categories [38]: (*a*) first-order intensity histogram statistics; (*b*) gray-level co-occurrence matrix features; (*c*) gray-level run length matrix; (*d*) gray-level size zone matrix; (*e*) gray-level dependence matrix; (*f*) neighboring gray tone difference matrix. The SFS algorithm has been exploited to select the most discriminating features, used to feed the XGBoost classifier.

### Predictive model development

The cohort of 237 patients selected at Center A (235 women, 2 men (age range: 17–89 years; mean age ± SD: 53.1 ± 15 years)) provided the 237 FBLs (size range: 4–90 mm; mean size ± SD: 15.1 ± 9.4 mm) which were used as a specific subset for the development of the predictive radiomics model. According to SOR, 137 out of 237 (57.8%) FBLs were benign and 100 out of 237 (42.2%) were malignant (Table 1).

Table 3 details the results obtained in the training phase, according to the two different input configurations. The association of US BI-RADS and radiomic features provided a statistically significant improvement for all the evaluated parameters when compared with radiomic analysis alone.

**Table 3:** Results obtained by the predictive model in the training phase

	Predictive model	
	Radiomic features	Radiomic features + US BI-RADS
Accuracy*	0.581 ± 0.099	0.933 ± 0.050
Specificity*	0.679 ± 0.126	0.947 ± 0.060
Sensitivity*	0.448 ± 0.170	0.914 ± 0.093
PPV*	0.505 ± 0.156	0.932 ± 0.072
NPV*	0.631 ± 0.086	0.942 ± 0.059
F1-score	0.474 ± 0.162	0.923 ± 0.081
AUROC*	0.581 ± 0.121	0.981 ± 0.025

Values are expressed as mean ± standard deviation

^*^P < 0.001

### External validation

The cohort of 115 patients (115 women, age range: 23–89 years; mean age ± SD: 51.6 ± 14.6 years) selected at Center B provided the 115 FBLs (size range: 3–50 mm; mean size ± SD: 14.7 ± 8.6 mm) which were used as a specific subset for the external validation of the predictive radiomics model. According to SOR, 70 out of 115 (60.9%) FBLs were benign and 45/115 (39.1%) were malignant (Table 1).

**Fig. 4:**
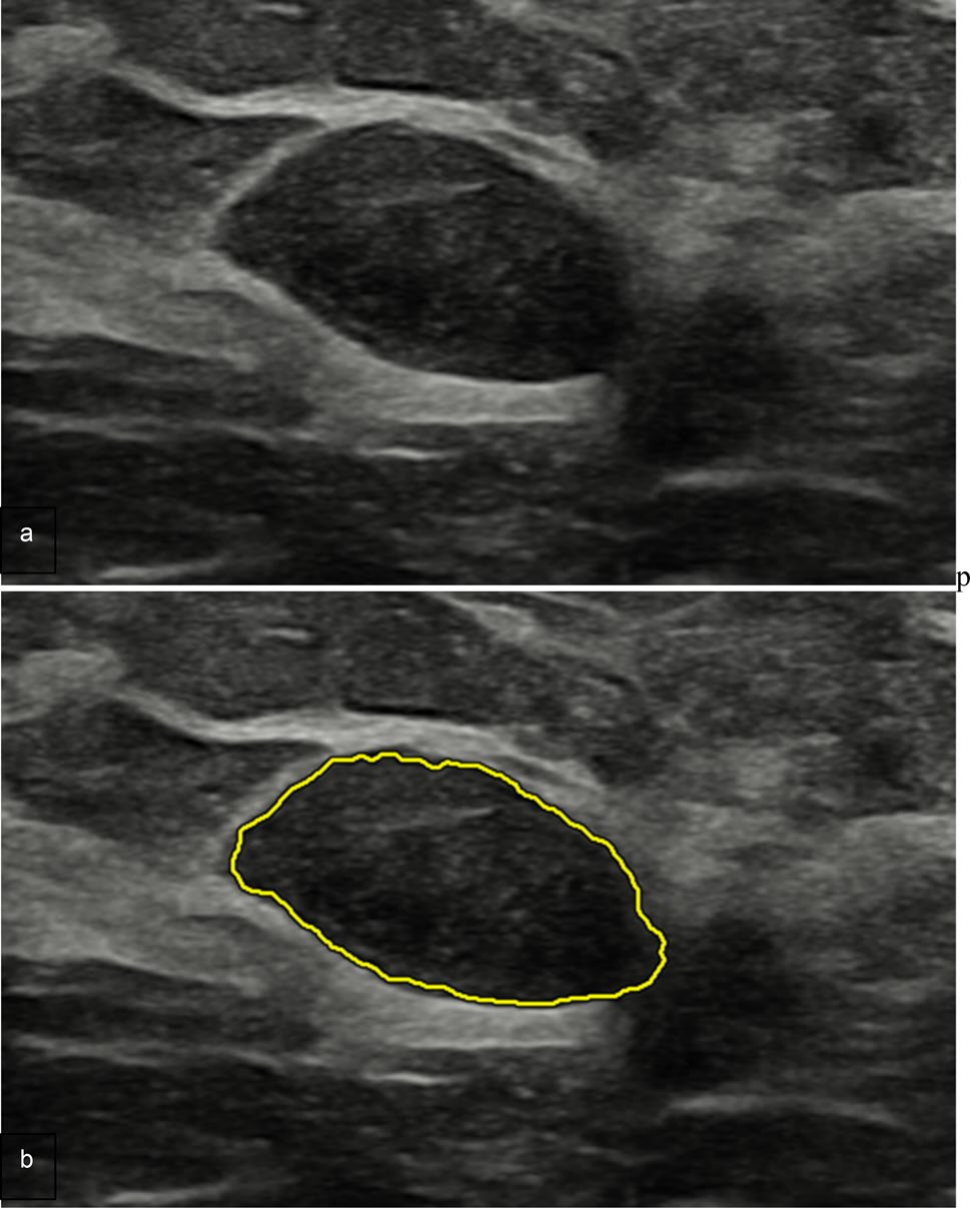
Luminal A ductal carcinoma in situ a 39-year-old woman with a palpable right breast lump on her lower inner quadrant. **a** B-mode US depicts a 2 cm hypoechoic focal breast lesion, oval-shaped, with parallel orientation and lobulated margins. **b** AI-based semi-automated lesion segmentation. US BI-RADS categorization was 3. US BI-RADS + radiomic features classification was: malignant

**Fig. 5:**
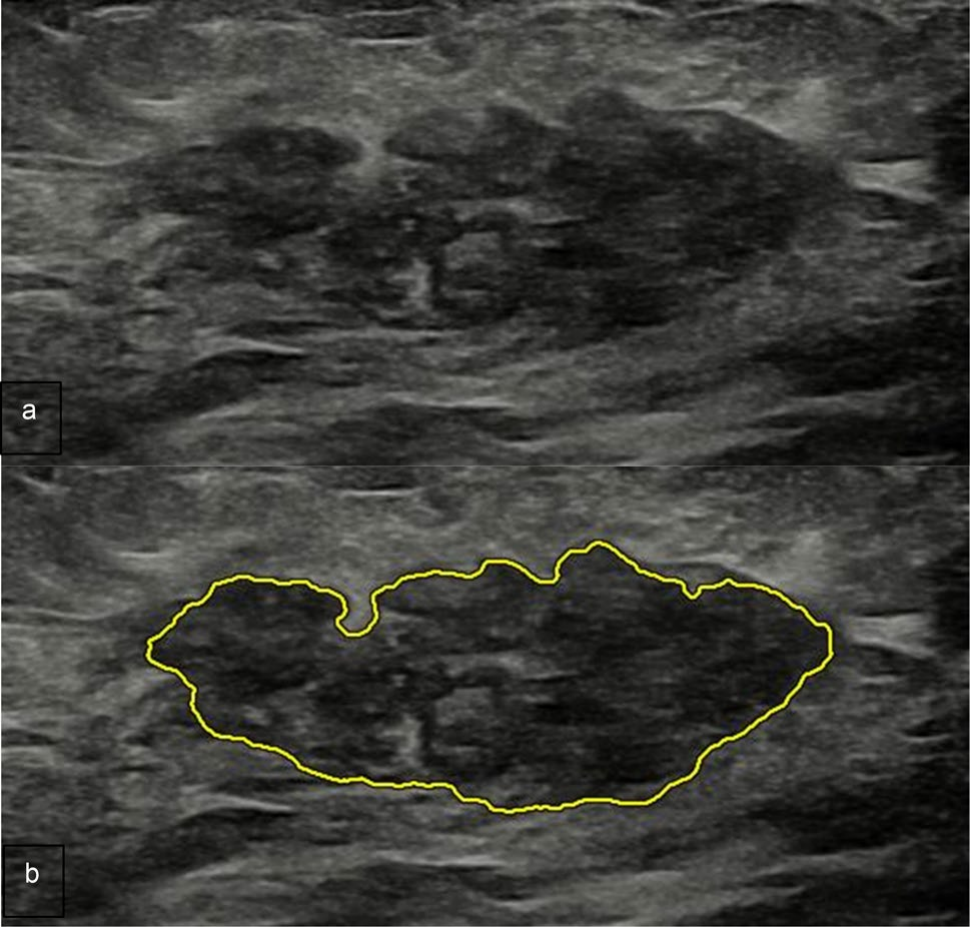
Fibroadenoma in a 35-year-old woman with a palpable left breast lump on her central upper quadrant. **a** B-mode US depicts a 3.4 cm hypoechoic focal breast lesion, oval-shaped, with parallel orientation and lobulated margins. **b** AI-based semi-automated lesion segmentation. BI-RADS categorization was 4. BI-RADS + radiomic features classification was: benign

**Fig. 6:**
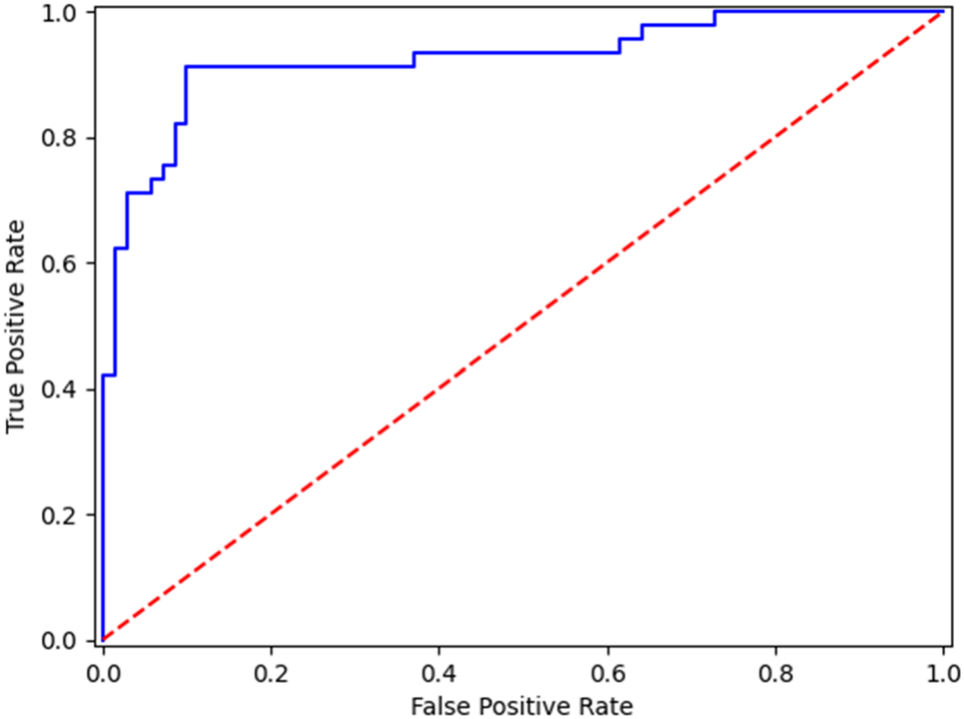
The ROC curve obtained in the validation phase considering the BI-RADS + radiomic features, with the AUROC = 0.925

Table 4 details the results obtained from the external validation dataset. The assessment made by the expert radiologist only, ML model only and the combination radiologists and ML model yielded sensitivity values of 0.933, 0.544, 0.756 specificity of 0.9, 0.6, 0.928, positive predictive values of 0.857, 0.416, 0.872 and negative predictive values of 0.955, 0.628, 0.855, respectively. Overall, the association of US BI-RADS and radiomic features yielded a not statistically significant improvement for the Specificity (*p* = *0.54*) (Fig. 4) with a statistically statistically significant decrease in Sensibility (*p* = *0.021*) (Fig. 5) when compared with US BI-RADS assessment only. Figure 6 details the ROC curve obtained in the validation phase considering the BI-RADS + radiomic features, with a corresponding AUROC of 0.925.

**Table 4:** Results obtained by the predictive model in the validation phase

	Predictive model		US BI-RADS [CI]	*p*-value
	Radiomic features	Radiomic features + US BI-RADS [CI]		
Accuracy	0.539	0.861 [0.786–0.913]	0.913 [0.847–0.952]	0.213 ^+^
Specificity	0.600	0.929 [0.866–0.963]	0.9 [0.832–0.943]	0.540 ^+^
Sensitivity	0.544	0.756 [0.670–0.825]	0.933 [0.872–0.966]	0.021*
PPV	0.416	0.872 [0.798–0.921]	0.857 [0.782–0.910]	0.839 ^+^
NPV	0.628	0.855 [0.779–0.908]	0.955 [0.900–0.980]	0.046*
F1-score	0.471	0.810 [0.731–0.867]	0.893 [0.826–0.938]	0.037*
AUROC	0.553	0.925 [0.862–0.988]	0.917 [0.866–0.968]	0.339 ^+^

Statistical significance: + not significant; *P < 0.05

### Explanation of the model prediction

**Fig. 7:**
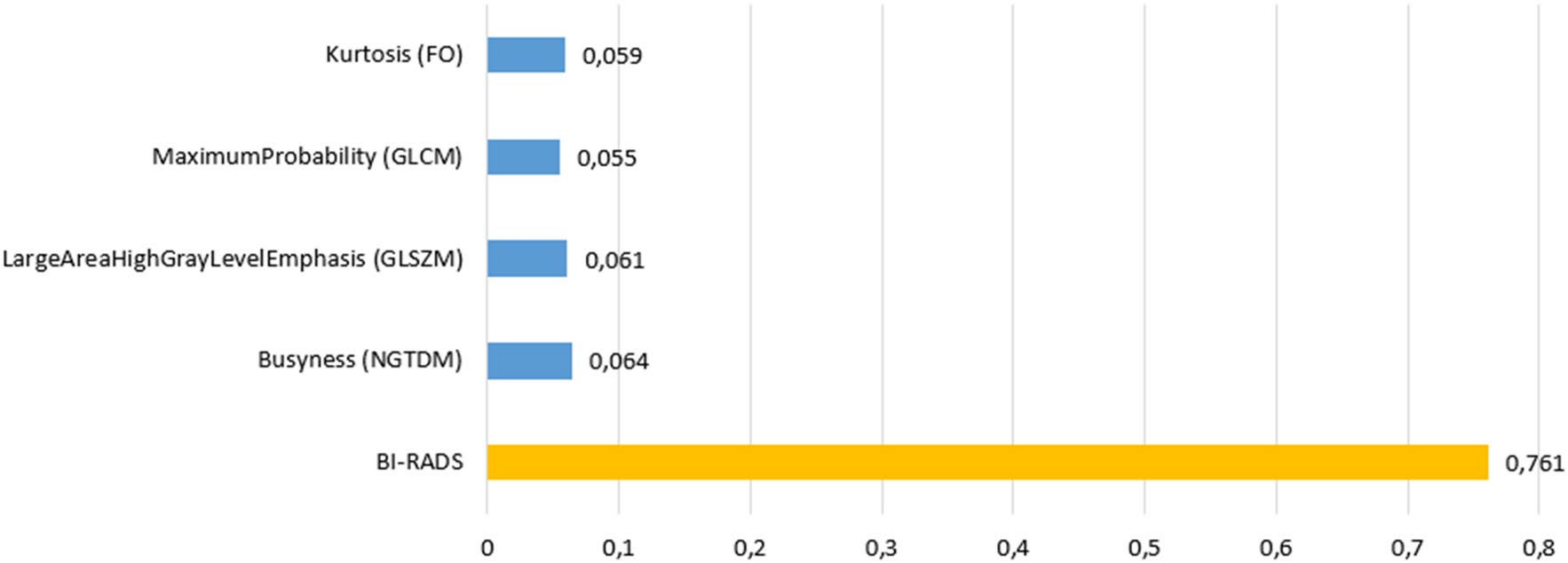
Bar diagram illustrating the importance of features in the prediction of the proposed model. In blue the radiomic features and in orange the BI-RADS

The BI-RADS was the most important feature for the model. However, the contribution of the radiomic features for the classification significantly increases the performance. Figure 7 details the relative importance that each feature has in the prediction of the XGBoost-based model.

The following four features were also found to be the most predictive: *Busyness*, (benign *vs.* malignant: 0.451 ± 0.056 *vs.* 0.460 ± 0.047; *p-value:* 0.008)), *LargeAreaHighGrayLevelEmphasis* (benign *vs.* malignant: 8927.511 ± 695.433 *vs.* 7892.846 ± 640.505; *p-value:* 0.011), *Kurtosis* (benign *vs.* malignant: 4.108 ± 0.156884 *vs.* 4.656 ± 0.306; *p-value:* 0.037) and *MaximumProbability* (benign *vs.* malignant: 0.054 ± 0.007 *vs.* 0.056 ± 0.008; *p-value:* 0.032).

## Discussion

The radiomic workflow proposed here extracts interpretable biomarkers and enables the development of predictive models that—exploiting shallow learning methods—manages better an analysis on tabular data. Radiomic biomarkers and shallow learning methods allow high-performance and interpretable models.

Although promising, ultrasound radiomics technologies for breast cancer assessment are currently under scrutiny, with reported AUC values in the differentiation between benign versus malignant breast masses ranging from 0.817 to 0.961 [14].

Lesion segmentation is the first and in many aspects the most crucial step of the radiomics process of feature extraction. On the one hand, a careful recognition of the breast lesion and its precise contouring is deemed mandatory. On the other hand, the segmentation task is performed by a dedicated operator, often a physician, it is time-consuming and operator-dependent, not to mention the introduction of a possible source of error [12]. In our study, we tried to overcome all these issues by using an AI software, which has been previously proved to improve the diagnostic performance of radiologists as well as intra- and inter-reader agreement in the characterization of FBLs [39]. This AI algorithm performs the lesion segmentation instantly, thus completely eliminating the relevant amount of time usually required to complete this task, at the same time leaving to the operator the final decision to accept the proposed segmentation.

In our experience, the semi-automated lesion segmentation process was highly reproducible, with a high rate of acceptance for both operators (91.3% and 93.3%) with no statistically significant difference in terms of discordance (*p-value: 0.515)*. Interestingly enough, the analysis of not accepted segmentations showed a prevalence of malignant lesions, which were characterized by a posterior shadowing at B-Mode US. Notably, a study encompassing 367 patients showed that the peripheral tissue around a breast lesion influences the subsequent classification based on B-mode images: paradoxically, when assessing US images of breast cancer by means of radiomics, the perfect separation between the lesion and the surrounding breast parenchyma might not be the best option [40].

In our series, the stand-alone radiomics model showed an AUC of 0.644. This finding is in line with a study evaluating four qualitative ultrasound features (regular tumor shape, no angular or spiculated margin, posterior acoustic enhancement and no calcification) in 252 breast lesions to predict the histological grade, Ki67, HER2, axillary lymph node metastasis and lymphovascular invasion, with corresponding AUC values of 0.673, 0.680, 0.651, 0.587 and 0.566, respectively [41]. Similarly, we did not include in our radiomics model any color Doppler or US elastography information, which might have improved the diagnostic performance, as reported in [42]. Furthermore, we implemented a machine learning model, whereas deep learning algorithms have been shown to be more proficient with a reported AUC of 0.955 in a study encompassing 10,815 and 912 multimodal and multiplane breast US images for training and prospective testing, respectively [43]. Noteworthy, in a prospective study including 124 women, quantitative multiparametric multimodal US provided the best diagnostic performance for breast cancer diagnosis [44].

In our validation cohort, when adding radiomic features to US BI-RADS classification made by the radiologist, we obtained an AUC of 0.925, which is within the range of previously reported data. Of note, we observed a not statistically significant increase in specificity (0.929 *vs*. 0.9, *p* = *0,540*) at the expense of a statistically significant decrease in sensitivity (0.756 *vs.* 0.933, *p* = *0,021*). In particular, the combined model allowed the detection of two more benign lesions (65 *vs.* 63, respectively) but missed eight breast cancers (34 *vs.* 42, respectively), with a corresponding decrease in detection rate from 93.3 to 75.6%. The observed reduction of false positive rate and the consequent decrease of the number of unnecessary biopsies, although not statistically significant, is in line with the study by Shen et al., who presented an AI system that achieved an AUROC of 0.976 on a test set consisting of 44,755 exams as well as a decrease in the false positive rates by 37.3% and reduction of requested biopsies of 27.8% while maintaining the same level of sensitivity [45]. Differently from this latter study, in our prospective study the impact of missed cancer due to the increase of false negative rate outweighed the decrease of the number of unnecessary biopsies allowed by the reduction of false positive rate. However, the retrospective nature of that study prompted the authors to ask for a prospective validation before it can be widely deployed in clinical practice.

Comparing our results to a recent retrospective study encompassing 201 FBLs, a ML classifier showed higher accuracy in comparison with an expert radiologist (82% *vs.* 79.4%) in the differential diagnosis of benign and malignant breast masses, but the difference was not statistically significant. (*p* = 0.815) [46]. Of note, in that study the radiologist was able to read only one defined US image per lesion, being unaware of the patient's clinical history and, most notably, being unable to perform a complete US scan. On the contrary, in our study the reading radiologist performed a proper US scan, whereas the radiomics model was based on still images, which may not fully depict the US features of a breast mass. Furthermore, the reading radiologist made the diagnosis on the basis of all the available data, thus more closely resembling real clinical practice. These circumstances may at least partially explain the higher sensitivity of the expert radiologist observed in our series in comparison with the radiomics model. The same limitations affect a study focusing on an automatic classification of ultrasound breast lesions using a deep convolutional neural network (dCNN) involving 582 patients [18]. In that study, the performance of dCNN was found to be comparable to that of two radiologists with more than 5-year experience (AUC of 83.8 for the dCNN *vs*. 86.9 and 82.3 for the humans on the internal dataset, respectively), but the authors stated that the final decision should be always left to the radiologists. This statement is supported by our results, taking in account that it is based on the real clinical practice of highly trained and expert breast radiologists.

Noteworthy, a multicenter study by Gu et al., encompassing US images of 5012 patients, found out a statistically significant decrease in sensitivity values in the external test cohort of a binary deep learning model in comparison with the original radiologists (89.81% *vs.* 99.30%, *p* = 0.0020) [47]. However, in the same study a six categories BI-RADS-based deep learning model achieved sensitivity values not statistically different from those of the reading radiologists (95.37% *vs.* 99.30%, *p* = 0.1250). This binary assessment was also present in our study, and might have negatively affected our radiomic model results.

From a value-based healthcare perspective, AI may provide useful data-driven tools to empower multimodality breast imaging in the setting of screening, lesion characterization, therapy guidance and monitoring assessment [48]. Nevertheless, a translational gap still exists. In the near future, the AI-based system in mammography and ultrasound is not expected to replace radiologists, but instead to offer support in the decision-making process or to reduce the radiologist’s workload [49, 50]. To this purpose, multiparametric MRI may provide information on pathophysiological tumor characteristics, useful for imaging biomarker research aimed at improving prediction of treatment response, disease-free survival, molecular subtype and lymph node status [51–53]*.* However, multiparametric MRI and other imaging modalities still need further efforts to fully exploit the AI and radiomics in order to better individualize breast cancer treatment in the era of precision medicine [48].

In our series, the most important radiomic features were *Busyness* (belonging to the NGTDM category) is a measure of the change from a pixel to its neighbor. A high value for busyness indicates a ‘busy’ image, with rapid changes of intensity between pixels and its neighborhood. Analysis of the two distributions (i.e., malignant, benign) shows that malignant cases are associated with higher busyness values than benign cases. This is symptomatic of the fact that malignant lesions generally exhibit a more heterogeneous pattern, where areas with different densities (e.g., habitat) are present within the lesion. Then, *LargeAreaHighGrayLevelEmphasis* (belonging to the GLSZM category) measures the proportion in the image of the joint distribution of larger size zones with higher gray-level values. In malignant cases, it is more likely to have regions of higher density (i.e., areas with high gray values). *Kurtosis* (belonging to the FO category) is a measure of the ‘peakedness’ of the distribution of values in the image ROI. A higher kurtosis implies that the mass of the distribution is concentrated toward the tail(s) rather than toward the mean. A lower kurtosis implies the reverse: that the mass of the distribution is concentrated toward a spike near the mean value. This trend is perfectly aligned with the clinical literature: benign lesions generally have a more oval (roundish) shape than malignant lesions, which have a wide and greater variability in shape. This aspect reflects the fact that malignant masses have greater kurtosis than benign ones [54, 55]. Finally, maximum probability (belonging to the GLCM category) calculates the occurrences of the most predominant pair of neighboring intensity values, thus revealing the existence of a regular pattern within the lesion.

Our study has limitations. Firstly, the prevalence of breast cancer in our study population is higher than that of the general population, leading to a selection bias. Actually, considering that the presence of unbalanced datasets leads a ML classifier to classify the most represented class better, to obtain a predictive model capable of accurately classifying two different classes, there is the need to have a balanced dataset (i.e., benign and malignant) [35]. Furthermore, as secondary referral centers for breast cancer, our population might present a higher number of malignancies in comparison with the general population.

Secondly, our dataset consisted of 352 patients but a larger sample might have granted a more robust ML assessment. In third place, we have not included color Doppler or elastosonography information into our radiomics model, nor clinical information: this information might have led to different values of diagnostic accuracy of the ML model. Further studies are needed to address this issue. Furthermore, though we assured the explainability of our machine learning system, a deep learning approach might be a future option for building more proficient radiomics models.

Classifiers based on shallow learning algorithms are an optimal starting point for the clinical domain since they can be easily interpreted and explained, but unfortunately, they cannot achieve the performance achievable by deep models, which, however, do not guarantee explainability. As future developments, there is the intention to implement more advanced and robust techniques to implement explainability in deep learning models.

In conclusion, our study showed that an artificial intelligence-based semi-automated lesion segmentation makes the extraction of ultrasound-derived radiomics features instantaneous, still maintaining high reproducibility. Posterior acoustic shadowing was the most important feature determining the unacceptance of semi-automated segmentation. In our experience, the combination of radiomics and US BI-RADS classification has led to a potential decrease in unnecessary biopsies. Nevertheless, in our series, a not negligible increase in potentially missed cancers was observed. To avoid such a negative effect, AI-based systems must reach adequate diagnostic performance so that they can be applicable to wide populations [56]. As a consequence, further larger international multicenter studies are needed before implementing radiomics features in the routine US assessment of breast cancer.

## Declarations

### Guarantor

The scientific guarantor of this publication is Prof. Tommaso Vincenzo Bartolotta.

### Statistics and biometry

Four of the authors have significant statistical expertise (DM, CM, FP, LR).
